# Adapting and Scaling a Digital Health Intervention to Improve Maternal and Child Health Among Ethnic Minority Women in Vietnam Amid the COVID-19 Context: Protocol for the dMOM Project

**DOI:** 10.2196/44720

**Published:** 2023-04-24

**Authors:** Bronwyn McBride, John O'Neil, Phuong Chi Nguyen, Dang Thuy Linh, Hue Thi Trinh, Nguyen C Vu, Liem T Nguyen

**Affiliations:** 1 Faculty of Health Sciences Simon Fraser University Burnaby, BC Canada; 2 Institute of Population, Health and Development Hanoi Vietnam

**Keywords:** mobile health, digital health, Vietnam, maternal health, maternal, newborn, and child health, MNCH, health equity, ethnic minority, maternal and child health, child health, ethnography, coronavirus, COVID-19, perinatal, antenatal, woman, mother, pregnancy, newborn, infant, baby, ethnic, ethnicity, visible minority, culture

## Abstract

**Background:**

Due to interconnected structural determinants including low maternal health knowledge, economic marginalization, and remoteness from low-capacity health centers, ethnic minority women in remote areas of Vietnam face severe maternal, newborn, and child health (MNCH) inequities. As ethnic minorities represent 15% of the Vietnamese population, these disparities are significant. mMOM—a pilot mobile health (mHealth) intervention using SMS text messaging to improve MNCH outcomes among ethnic minority women in northern Vietnam—was implemented from 2013-2016 with promising results. Despite mMOM’s findings, exacerbated MNCH inequities, and digital health becoming more salient amid COVID-19, mHealth has not yet been scaled to address MNCH among ethnic minority women in Vietnam.

**Objective:**

We describe the protocol for adapting, expanding, and exponentially scaling the mMOM intervention *qualitatively* through adding COVID-19–related MNCH guidance and novel technological components (mobile app and artificial intelligence chatbots) and *quantitatively* through broadening the geographical area to reach exponentially more participants, within the evolving COVID-19 context.

**Methods:**

dMOM will be conducted in 4 phases. (1) Drawing on a review of international literature and government guidelines on MNCH amid COVID-19, mMOM project components will be updated to respond to COVID-19 and expanded to include a mobile app and artificial intelligence chatbots to more deeply engage participants. (2) Using an intersectionality lens and participatory action research approach, a scoping study and rapid ethnographic fieldwork will explore ethnic minority women’s unmet MNCH needs; acceptability and accessibility of digital health; technical capacity of commune health centers; gendered power dynamics and cultural, geographical, and social determinants impacting health outcomes; and multilevel impacts of COVID-19. Findings will be applied to further refine the intervention. (3) dMOM will be implemented and incrementally scaled across 71 project communes. (4) dMOM will be evaluated to assess whether SMS text messaging or mobile app delivery engenders better MNCH outcomes among ethnic minority women. The documentation of lessons learned and dMOM models will be shared with Vietnam’s Ministry of Health for adoption and further scaling up.

**Results:**

The dMOM study was funded by the International Development Research Centre (IDRC) in November 2021, cofacilitated by the Ministry of Health, and is being coimplemented by provincial health departments in 2 mountainous provinces. Phase 1 was initiated in May 2022, and phase 2 is planned to begin in December 2022. The study is expected to be complete in June 2025.

**Conclusions:**

dMOM research outcomes will generate important empirical evidence on the effectiveness of leveraging digital health to address intractable MNCH inequities among ethnic minority women in low-resource settings in Vietnam and provide critical information on the processes of adapting mHealth interventions to respond to COVID-19 and future pandemics. Finally, dMOM activities, models, and findings will inform a national intervention led by the Ministry of Health.

**International Registered Report Identifier (IRRID):**

PRR1-10.2196/44720

## Introduction

### Maternal, Newborn, and Child Health Inequities Among Ethnic Minority Women in Vietnam

Despite Vietnam’s remarkable progress in improving maternal, newborn, and child health (MNCH) outcomes during the era of the Millennium Development Goals, increasing evidence demonstrates that ethnic minority communities living in remote areas have not benefited equally from this progress—MNCH inequities between the Kinh (ethnic majority group in Vietnam) and ethnic minority communities have in fact widened [1-3]. There are 54 ethnic groups in Vietnam, with the Kinh majority representing 85% (98 million) of the national population and 53 ethnic minority groups representing the remaining 15%. In Vietnam, as in other Southeast Asian countries, the term “ethnic minority” is commonly utilized by government bodies for populations who may elsewhere be classified as Indigenous peoples. As government authorities in Vietnam do not recognize Indigenous identity in programs or reports, this paper utilizes the terminology “ethnic minority.”

Among ethnic minority populations, 75% live in 13 provinces in the northern mountain and central highlands regions, which are known for high concentrations of poverty [3]. Ethnic minority populations in Vietnam have higher total fertility rates, adolescent birth rates, and infant and child mortality rates and lower life expectancies [1,4,5]. An analysis of data from 4 recent censuses of Vietnam showed that at all survey time points (2000, 2006, 2011, 2014), the proportions of women who received skilled antenatal care and who gave birth with assistance from skilled staff were significantly higher among Kinh, higher economic status, and urban populations, highlighting clear structural inequities in health access for ethnic minority women in remote settings [2]. Vietnam’s overall progress on MNCH indicators obscures severe disparities among marginalized ethnic minorities: in 2020-2021, 95% of all live births in Vietnam were delivered by a skilled birth attendant, yet this figure was only 37.7% among Hmong minority women [4]. Data from the same 2020-2021 national survey show that the percentage of women who received at least four antenatal care visits during pregnancy was 88.2% among the Kinh and Hoa, 62.3% among the poorest quintile, and 10.6% among the Hmong [4].

Shaped by decades of structural deprivation and social exclusion, specific factors contributing to the profound MNCH disparities faced by ethnic minority populations are limited access to education, economic marginalization, remote and rural residence (where there are fewer and lower capacity health centers and a documented shortage of health workers [5,6]), limited access to MNCH information, low reproductive health knowledge, language barriers, and poor access to and uptake of perinatal care services [2,3,5,7,8]. A 2021 study found that economic marginalization and living over 5 km from the nearest health facility were associated with having fewer than 4 antenatal care visits [7]. Importantly, documented inequities along ethnic lines in health care seeking and utilization, MNCH, nutrition, and oral health and hygiene suggest that minority ethnicity alone is a critical determinant impacting health outcomes [9]. Taken together, this evidence highlights how the confluence of ethnic minority status and related facets of socioeconomic exclusion has shaped severe and intractable MNCH inequities among ethnic minority women in Vietnam.

### mMOM 2013-2016

To address MNCH disparities among ethnic minority women in remote areas, the “mMOM” project (2013-2016) piloted and evaluated a low-cost mobile health (mHealth) intervention in Thai Nguyen province, northern Vietnam. mMOM was developed by the Institute for Population, Health and Development in Hanoi (PHAD) and Simon Fraser University (SFU) in Vancouver and funded by the International Development Research Centre (IDRC) from Canada. The project aimed to determine whether integrated use of an electronic health information system, low-cost mobile technology, a user-provider interaction model, and behavior change communication (BCC) via SMS text messaging could improve access to MNCH information and services among perinatal ethnic minority women and their families. Developed using templates from the Mobile Alliance for Maternal Health [10] and adapted for local acceptability, mMOM administered 2 SMS text messaging programs for pregnancy and postpartum periods, featuring information, education, and communication (IEC); reminder; and BCC messages designed to support maternal health and to promote deeper interaction between ethnic minority women and the health care system. Participants were registered in mMOM according to their specific week in pregnancy/new motherhood and received tailored information on healthy fetal and infant development 2-3 times weekly via SMS text messaging. Additional details about the mMOM project development and protocol are available elsewhere [11,12].

Between 2013-2016, the mMOM intervention built and implemented an integrated mHealth platform, which sent timely IEC, reminder, and BCC text messages to 961 participating women in 8 communes in Thai Nguyen toward stimulating ethnic minority women’s demand for quality perinatal care. In midterm and final qualitative evaluations assessing mMOM’s acceptability and impact involving over 60 participants, women expressed satisfaction with the intervention’s quality, timeliness, and convenience, and health workers reported increased efﬁciency and quality of care [12]. The mMOM project increased care seeking from ethnic minority women and promoted increased contact and strengthened relationships between participants and health providers, demonstrating the potential of mHealth to address the marginalization of ethnic minority women from the health system in this setting [12].

### Need for the Current Study

#### Broader Geographic Area

Despite these findings and mMOM’s promise in promoting MNCH among structurally marginalized ethnic minority women, the intervention’s impacts were limited due its pilot nature and implementation in only 8 communes in a single province in Vietnam. Implementing an adapted and scaled version of the intervention across a broader geographic area, thus reaching more participants, is necessary to generate robust empirical evidence on the potential of mHealth to improve MNCH health equity in Vietnam and to build models that can be replicated and utilized for country-level mHealth projects.

#### SMS Text Messaging Versus Mobile App

Despite the high penetration of mobile services in Vietnam (143 mobile subscriptions for every 100 people in 2020 [13]), the application of mHealth for MNCH in Vietnam remains limited, with the exception of a current notable study examining a mobile app to promote early initiation and exclusive use of breastfeeding [14]. In alignment with this current research, given the rapid expansion of telecommunication technology and coverage of 4G network ever since mMOM was completed in 2016 and extensive smartphone uptake and usage even in remote areas [15], the potential impacts of mobile app–based MNCH delivery (rather than SMS text messaging based) among ethnic minority women must also be investigated and compared.

#### COVID-19, Exacerbated Health Inequities, and the Salience of mHealth

Current gaps in mHealth research and utilization in Vietnam were rendered even more salient by COVID-19, which exacerbated existing inequities in health access and outcomes globally—particularly for communities already marginalized from health systems including ethnic minority populations, women, immigrants, refugees, and people in rural/remote areas [16-20]. The pandemic underscored the urgency of research and development surrounding digital health interventions in the context of physical distancing restrictions and overloaded health facilities to maintain health access and prevent deepening health disparities amid COVID-19. The pandemic impacts have also been gendered and intersectional: globally, perinatal women represent a vulnerable population who have faced poorer maternal health outcomes and increased mental health concerns including heightened postpartum depression amid COVID-19 [21,22], and these disparities have been shaped by economic and class status, ethnicity, and geographic location [17,19,22-26]. This emerging evidence and calls for virtual and digital health programming to support perinatal women during the pandemic [21] suggest an urgent need for mHealth interventions that support maternal mental health and broader MNCH in Vietnam through the evolving COVID-19 crisis and beyond.

### Objective

Despite mMOM highlighting the potential of mHealth to promote MNCH among ethnic minority women in Vietnam, existing severe MNCH inequities among ethnic minority women, and digital health approaches becoming even more salient amid COVID-19, mHealth has not yet been leveraged or scaled to address MNCH in Vietnam. Thus, the objective of this paper is to describe the protocol for adapting, expanding, and exponentially scaling the mMOM intervention *qualitatively* through the addition of COVID-19–related MNCH guidance and novel technological components (a mobile app and the use of artificial intelligence [AI]) and *quantitatively* through its expansion over a broader geographical area to reach exponentially more participants. This protocol details the adaptation of the 2013-2016 mMOM intervention (SMS text messaging intervention reaching 8 communes in 1 district with 900 participants) to the dMOM intervention (SMS text messaging, mobile app, and AI intervention reaching 71 communes in 5 districts with 9000+ participants), within the unique pandemic context.

## Methods

### Overview

The dMOM project will be conducted in 4 phases: (1) updating, adapting, and expanding the mMOM intervention; (2) scoping study and rapid ethnographic fieldwork; (3) dMOM intervention implementation and incremental scaling; and (4) intervention evaluation and documentation of lessons learned. A series of smaller studies conducted through project phases 2-4 will gather evidence on different dMOM facets and models to inform replication and scaling at the country level.

### Phase 1: Updating, Adapting, and Expanding the Intervention

For phase 1, the mMOM project components will be (1) updated and adapted to respond to COVID-19 and its impacts and (2) expanded to include a mobile app and AI chatbots to more deeply engage participants. To adapt the intervention to serve perinatal women’s unique vulnerabilities and needs through the pandemic and beyond, a literature review of publications on MNCH amid COVID-19 will be undertaken by a multidisciplinary team. Institutional and governmental guidelines on pregnancy during COVID-19 as well as peer-reviewed manuscripts, conference abstracts, and case studies will be screened and reviewed, with relevant information extracted to inform, revise, and update the original mMOM intervention content. As a result, the IEC, reminder, and BCC messages in the SMS text messaging packages and the mobile app content will include critical information (i.e., on COVID-19 risk awareness, testing, vaccinations, and maternal mental health) to support healthy pregnancies and newborn care during the pandemic and postpandemic contexts and ensure that the dMOM intervention reflects international and country-level institutional guidance on MNCH during COVID-19. Particular attention will be paid to emerging evidence and institutional guidelines surrounding COVID-19, mental health, and postpartum depression among perinatal women [21,22,27]. Finally, the mMOM BCC messages will be updated to target not only ethnic minority women and women living in remote mountainous areas, but also perinatal women in less remote areas, given the novel constraints in MNCH care accessibility and isolation from health services engendered by the pandemic.

To offer participants a more nuanced, customizable, and lower-cost avenue for engaging with the intervention, the dMOM app will be developed in partnership with computing science consultants to leverage smartphone capabilities and provide a more complex variety of functions to improve MNCH access and health outcomes among perinatal ethnic minority women. Similar to the mMOM SMS text messaging intervention, the dMOM mobile app will provide person-centered, tailored, and timely information according to women’s specific stage of pregnancy/new motherhood. However, the dMOM app will feature more information per message/interaction (messages will not be subject to SMS text messaging character limits); photo, audio, and video messages to increase engagement with participants; and audio-photo messages as an important accessibility consideration for women with limited literacy and/or disabilities. The dMOM app also will reduce the cost of project participation (there are no associated costs after app installation, unlike SMS text messaging fees) and support health promotion activities among village health workers through the digitalization of traditional, familiar, and culturally acceptable IEC materials (i.e., MNCH posters and educational cartoons) and their inclusion in the app to reach a broader audience.

An assessment of how AI chatbots (automatic dialogue system) can be leveraged in dMOM to promote MNCH care and support project scalability and sustainability will be conducted through a literature review and consultation with AI experts who are members of the research team, and relevant components of existing uses of AI for MNCH will be extracted and adapted for use in the dMOM app. AI chatbots will be developed and piloted, with information collected to train AI models to correct SMS text messages, assess MNCH risks, and provide advice that is responsive to women’s inquiries and enables them to quickly act in a health emergency situation requiring expertise from a health professional. dMOM will utilize the richer technological capacities offered by a mobile app and AI chatbots and evaluate their effectiveness in improving the quality of MNCH BCC.

### Phase 2: Scoping Study and Rapid Ethnographic Fieldwork

For phase 2, a broad scoping study using mixed methods and rapid ethnographic fieldwork will be conducted at the project outset to involve key stakeholders in the intervention’s codevelopment from its initial stages. The scoping study will begin with preliminary engagement meetings with the Ministry of Health, provincial and district health departments, women’s health service providers, community advocacy groups for women and ethnic minority populations, local authorities, village chiefs, and individual women to ensure that the intervention is shaped by key institutional and community voices.

The scoping study will apply mixed research methods including in-depth interviews; focus group discussions led by trained project staff; and survey questionnaires with pregnant and postpartum ethnic minority women, family members, and health workers at diverse levels. It will assess (1) unmet MNCH needs among ethnic minority women and their newborns; (2) roles of husbands, family members, and broader community members as well as their gendered power dynamics impacting MNCH care; (3) existing MNCH communication mechanisms, gaps, and MNCH health service utilization; (4) the extent of mobile phone use of ethnic minority women in the new districts in Thai Nguyen and Dien Bien provinces (i.e., districts not covered in the mMOM project) and social and cultural factors impacting phone ownership and usage; (5) literacy and language barriers to receiving SMS text messages and using apps; (6) technical capacity of commune health workers and health centers surrounding mHealth utilization and technical constraints (i.e., power outages and mobile network coverage); and (7) long-term, multilevel impacts of COVID-19 on MNCH access and outcomes, ethnic minority health, and broader socioeconomic status of remote communities. The study will also include a rapid ethnographic review of gendered determinants; ethnicity-based barriers; and other key social, cultural, economic, and political factors that may hinder or facilitate project outcomes, as well as in-depth interviews with other potential stakeholders (i.e., corporate and technological institutions and government bodies) at district and provincial levels to explore partnership opportunities and policy constraints to project implementation and scaling up.

Within the broader scoping study, 2 smaller intersectionality and digital health uptake studies will take place. The intersectionality study will explore ethnicity, gendered power relationships, geographical and social distancing, and other interconnected determinants of MNCH outcomes and mHealth applicability, not in isolation but how they interact dynamically with one another to shape severe health disparities among ethnic minority women in northern Vietnam [28,29], particularly as impacted by COVID-19. This intersectionality study will utilize a participatory action research and people-centered approach—identified as critical contributing factors to the uptake and success of mMOM [12]—toward codeveloping and continually refining the intervention in interaction with community members to ensure that it is culturally sensitive, sustainable, and responsive to community needs [30]. The digital health uptake study will explore digital health accessibility and acceptability in geographically and/or socially remote settings, especially among ethnic minority women. Given current research gaps on digital health preferences among ethnic minority groups, the study will investigate participants’ preferences for either the mobile app or SMS text messaging intervention; factors at individual, family, and societal levels informing their preferences; and potential solutions to increase uptake among ethnic minority women in remote areas toward reducing digital health inequity.

### Ethics Approval

Trained commune health staff will obtain oral informed consent from focus group and in-depth interview participants prior to their participation in the scoping study. The study has been approved by an institutional review board at PHAD, which is registered with and uses ethical protocols of the Office for Human Research Protections of the United States Department of Health and Human Services (2023/PHAD-DMOM-01). Project data are subject to privacy and security protocols of the Office for Human Research Protections. Participants in the focus groups and in-depth interviews will be remunerated 100,000 Vietnamese Dong (~US $4.20) for their expertise, time, and travel.

The scoping study’s findings will be used to refine the scope of work, project activities (e.g., modifying the research design, updating intervention content, and selecting additional ethnic minority languages), and timeline in alignment with implementation capacities at the village and commune health center levels. The study’s involvement of stakeholders in shaping the dMOM intervention from the project outset is expected to increase their interaction with the project and empower and strengthen ethnic minority women and their families’ increased engagement in MNCH care [30]. It will also provide a critical opportunity for stakeholders to express COVID-19–related concerns regarding the intervention’s operation so that these concerns may be accounted for and mitigated.

### Phase 3: Intervention Implementation and Incremental Scaling

Upon completion of the first 2 study phases (updating and adapting the intervention content, developing the mobile app, and undertaking the scoping study), for phase 3, the dMOM intervention will be implemented and incrementally scaled. As the project plan will be informed by, updated, and revised based on the scoping study to ensure the intervention’s accessibility and acceptability to the target population, the below protocol represents only a general guideline. Implementation will be initiated with project staff conducting a training-of-trainers activity to (1) train commune health workers as dMOM project implementors including on health management information system operations and dMOM integration and (2) enable them to train village health workers in the intervention’s routine operation (i.e., registering participants, using the mobile app, and operating and monitoring the dMOM system). First, the updated SMS text messaging intervention will be implemented in Dinh Hoa, Thai Nguyen (the original mMOM intervention site) to initiate the intervention and troubleshoot potential challenges related to human resources, project staff training, and health management information system operation. This initial implementation period limited within 1 district will also provide critical ongoing engagement and continuity of the intervention while the dMOM app is being developed and ease the introduction of the dMOM app. Subsequently, the dMOM mobile app will be piloted in Dinh Hoa and 2 new districts in Thai Nguyen province (one remote and one urban district—Vo Nhai district and Song Cong city) and 2 districts (Muong Cha district and Dien Bien Phu city) within the even more remote and mountainous Dien Bien province, where ethnic minorities constitute 82.6% of the population [31]. To compare the relative acceptability, uptake, and MNCH impact of the dMOM mobile app to the SMS text messaging intervention, an equal number of communes in each district will be randomly assigned with the use of either the dMOM app or mMOM SMS text messaging to assess each component’s feasibility among participants. Although the 2013-2016 pilot mMOM intervention reached 961 perinatal women across 8 communes in 1 district, the dMOM intervention will be implemented across 71 communes in 5 districts of 2 provinces, aiming to reach approximately 9000 women participants over a 30-month period.

### Phase 4: Intervention Evaluation and Documentation of Lessons Learned

For phase 4, dMOM will be evaluated to assess whether SMS text messaging delivery or mobile app delivery engenders the most improved MNCH health-seeking behaviors and outcomes among ethnic minority women. The dMOM evaluation will involve data collection via multiple channels. Demographic and socioeconomic data will be collected by commune health staff at the intervention onset intervention when participants are registered. Monitoring and evaluation indicators on health-seeking behaviors and pregnancy and birth outcomes will be collected via automatic individual data collection and questions posed to participants within the dMOM app. The SMS text messaging and app systems will also be mined for back-end information regarding user statistics. Finally, qualitative in-depth interviews and quantitative survey questionnaires will be administered online or in-person, depending on COVID-19–related factors and feasibility. Data from these sources will be analyzed using thematic analysis in NVivo for qualitative data and regression models in Stata (StataCorp) or R (R Foundation for Statistical Computing) for quantitative data and triangulated to examine how they expand upon one another and explore both intended and potential unexpected outcomes of the intervention, aiming to improve the credibility, integrity, and utility [32] of the research to multilevel policy institutions in Vietnam and internationally. The documentation of lessons learned will be conducted and shared with Vietnam’s Ministry of Health and the public for adoption and further scaling up; upon project completion, the models developed and refined in dMOM will inform a national mHealth project led by the Ministry of Health, representing a scaling of components of the dMOM project to the country level.

During the evaluation phase, a smaller scalability study will draw on the data collected as part of all dMOM components and assess different scenarios associated with several scaling-up strategies. This study will identify factors that may facilitate or constrain effective and ethical scaling of dMOM models and will investigate the potential of the private sector as a financing mechanism to sustain a broader mHealth intervention for MNCH.

## Results

The dMOM study was funded by the IDRC in November 2021, cofacilitated by the Ministry of Health, and is being coimplemented by provincial health departments in the 2 mountainous provinces of Thai Nguyen and Dien Bien. The intervention received approval from and completed a memorandum of understanding with Vietnam’s Ministry of Health in May 2022. Phase 1 (literature review and updating, adapting, and expanding intervention components) was initiated in May 2022, and phase 2 (scoping study and rapid ethnographic fieldwork) is planned for December 2022. The entire study is expected to be complete at the end of June 2025.

## Discussion

### Expected Findings

This paper describes the protocol for adapting and exponentially scaling the mMOM intervention *qualitatively* through the addition of novel technological components (i.e., mobile app and the use of AI chatbots); *quantitatively* through its expansion over a broader geographical area; and in its *scope* through deep, ongoing engagement with the Ministry of Health toward the replication of the dMOM models at the country level. This protocol details the expansion of the pilot mMOM SMS text messaging intervention in 8 communes with 900 participants to an SMS text messaging, mobile app, and AI intervention in 71 communes reaching 9000+ participants.

This research will contribute to bridging current gaps in mHealth research and utilization in Vietnam, which were rendered even more salient by COVID-19 and exacerbated existing inequities in health access particularly for ethnic minority populations and people in rural/remote areas globally [16-20]. Our protocol and study aim to respond to research calls for virtual and digital health programming to support perinatal mental health and broader MNCH in Vietnam through the evolving COVID-19 crisis and beyond [21]. Through the dMOM intervention’s development, implementation, and evaluation of a mobile app for MNCH amid COVID-19, the project aims to contribute to urgently needed evidence on the application of digital health during the pandemic context, particularly in maintaining health access and promoting health equity among women, ethnic minority populations, remote communities, and other intersecting populations globally who are the most vulnerable to the negative socioeconomic and health impacts of pandemics.

### Limitations

Although this study will be conducted within a broader geographical area than the pilot mMOM study, it will only involve ethnic minority populations who are in contact with the health system, as commune health workers will only recruit women who present at the health center. As such, it is possible that the project may exclude members of the target population who are not known to health workers due to extremely remote residence, poverty, or language barriers.

### Study Implications

The outcomes and findings of the dMOM study will be relevant to future research on the applicability and use of mHealth and AI for MNCH for ethnic minority communities and for improved MNCH and health-seeking behaviors among other structurally marginalized populations and populations living in remote settings.

### Conclusions

This paper outlines the development of the dMOM intervention and its planned expansion and scaling amid the COVID-19 context. The dMOM research outcomes will generate important empirical evidence on the effectiveness of leveraging digital health to address intractable MNCH inequities among ethnic minority women and women living in remote areas and low-resource settings in Vietnam, build models for utilization and replication in country-level mHealth projects, and provide critical information on the processes of adapting mHealth interventions to respond to COVID-19 and other future pandemics.

## Abbreviations

AI: artificial intelligence
BCC: behavior change communication
IDRC: International Development Research Centre
IEC: information, education, and communication
mHealth: mobile health
MNCH: maternal, newborn, and child health
PHAD: Institute for Population, Health and Development
SFU: Simon Fraser University

## Data Availability

Data sharing is not applicable to this article, as no data sets were generated or analyzed during the current protocol study.
